# Quality Indicators for Safe Medication Preparation and Administration: A Systematic Review

**DOI:** 10.1371/journal.pone.0122695

**Published:** 2015-04-17

**Authors:** Marian Smeulers, Lotte Verweij, Jolanda M. Maaskant, Monica de Boer, C. T. Paul Krediet, Els J. M. Nieveen van Dijkum, Hester Vermeulen

**Affiliations:** 1 Department of Quality Assurance and Process Innovation, Academic Medical Center, Amsterdam, the Netherlands; 2 Emma Children’s Hospital, Academic Medical Center, Amsterdam, the Netherlands; 3 Department of Clinical Epidemiology, Biostatistics and Bioinformatics, Medical Faculty, Academic Medical Center and University of Amsterdam, Amsterdam, the Netherlands; 4 Department of Hospital Pharmacy, Amsterdam, Academic Medical Center, the Netherlands; 5 Department of Internal Medicine, Amsterdam, Academic Medical Center, the Netherlands; 6 Department of Surgery, Amsterdam, Academic Medical Center, the Netherlands; 7 Department of Nursing, the Amsterdam School of Health Professions, Amsterdam, the Netherlands; University of Catania, ITALY

## Abstract

**Background:**

One-third of all medication errors causing harm to hospitalized patients occur in the medication preparation and administration phase, which is predominantly a nursing activity. To monitor, evaluate and improve the quality and safety of this process, evidence-based quality indicators can be used.

**Objectives:**

The aim of study was to identify evidence-based quality indicators (structure, process and outcome) for safe in-hospital medication preparation and administration.

**Methods:**

MEDLINE, EMBASE and CINAHL were searched for relevant studies published up to January 2015. Additionally, nine databases were searched to identify relevant grey literature. Two reviewers independently selected studies if (1) the method for quality indicator development combined a literature search with expert panel opinion, (2) the study contained quality indicators on medication safety, and (3) any of the quality indicators were applicable to hospital medication preparation and administration. A multidisciplinary team appraised the studies independently using the AIRE instrument, which contains four domains and 20 items. Quality indicators applicable to in-hospital medication preparation and administration were extracted using a structured form.

**Results:**

The search identified 1683 studies, of which 64 were reviewed in detail and five met the inclusion criteria. Overall, according to the AIRE domains, all studies were clear on purpose; most of them applied stakeholder involvement and used evidence reasonably; usage of the indicator in practice was scarcely described. A total of 21 quality indicators were identified: 5 structure indicators (e.g. safety management and high alert medication), 11 process indicators (e.g. verification and protocols) and 5 outcome indicators (e.g. harm and death). These quality indicators partially cover the 7 rights.

**Conclusion:**

Despite the relatively small number of included studies, the identified quality indicators can serve as an excellent starting point for further development of nursing specific quality indicators for medication safety. Especially on the right patient, right route, right time and right documentation there is room future development of quality indicators.

## Introduction

Medication safety is an important topic because medication errors (MEs) are a common, serious and expensive type of medical error [1]. According to the NCCMERP a ME is defined as 'any preventable event that may cause or lead to inappropriate medication use or patient harm while the medication is in the control of the health care professional, patient or consumer’[2]. The medication process is vulnerable and error prone as it is a complex five phase process: (a) prescribing, (b) verifying, (c) preparing/dispensing (d) administering, and (e) monitoring. A ME can originate in all of these phases.

Rates of medication errors vary, depending on the detection method used. Estimates of the incidence of MEs vary between 5% and 25% of all medication administrations [3–6]. One-third of all MEs causing harm to patients in hospitals occur in the medication preparation and administration phase, which is predominantly a nursing activity [3, 7]. In some settings, medication orders are transcribed, dispensed, and then delivered for nurse administration. In other circumstances and settings, nurses are involved in both the dispensing and preparation of medications (in a similar role to pharmacists), such as crushing pills and preparing syringes with the correct dose. To improve medication safety, the medication process has to be assessed. A widely used method to do this is with quality indicators (QIs) [4, 8–10]. Quality indicators are explicitly defined and measurable items referring to the structure, processes, or outcome of care [9, 11]. The structure is the environment in which health care is provided, and includes material and health resources, operational factors, and organizational characteristics of the health care facility. The process is the method by which health care is provided and includes the giving and receiving of care by the practitioners and health care system. The outcome is the consequence of health care and includes the health status of patients. Quality indicators are usually described with a numerator, a denominator, and a performance standard. After assessment of the quality of care, quality indicators are used to measure whether improvement actions have the desired result as well as to monitor performance.

In order to use quality indicators to assess care in terms of medication preparation and administration, we must first define good quality and safe care. To ensure safe medication preparation and administration, nurses are trained to practice the “7 rights” of medication administration: right patient, right drug, right dose, right time, right route, right reason and right documentation [12, 13]. However, adhering to these 7 rights is not just the responsibility of the individual nurse, but also of the health care organization, i.e. the system within which the nurse functions. An ISMP editorial states that “The 7 rights are broadly stated goals or desired outcomes of safe medication practices for which organizations must accept responsibility and design failsafe ways that they can be achieved” [14]. A good example of this is the organization policy to remove concentrated electrolytes from patient care areas. When a ME occurs, this is often due to breach of one of the 7 rights. Therefore in this review we aimed to identify evidence-based quality indicators (structure, process and outcome) for the 7 rights of safe in-hospital medication preparation and administration.

## Methods

We conducted and reported this study according to the Preferred Reporting Items for Systematic Reviews and Meta-Analysis (PRISMA) statement (S1 Appendix)[15].

### Search strategy

To identify relevant studies, we systematically searched three electronic databases: MEDLINE, EMBASE and CINAHL. These databases were searched from their start date to January 19, 2015; no limitations on language or year of publication were applied. The search terms used combined keywords and medical subject headings for medication safety and quality indicators. We used the following search terms: patient safety, medication safety, medication error, medication administration, quality indicators, quality of care, nurses and nursing care (S2 Appendix for full searches of the databases).

In addition, we conducted a grey literature search in nine sources (search term used: indicators medication safety) to identify studies not indexed in the databases listed above:

- Institute for Healthcare Improvement at www.ihi.org.
- Agency for Healthcare Research and Quality (AHRQ) at www.ahrq.gov.
- The Joint Commission International at www.jointcommissioninternational.org.
- Open Grey at www.opengrey.eu.
- ProQuest at www.proquest.com.
- National Health Services at www.nhs.uk.
- The Australian Commission on Safety and Quality in Healthcare at www.safetyandquality.gov.au.
- The Canadian Patient Safety Institute at www.patientsafetyinstitute.ca.
- The National Institute of Health and Care Excellence at www.nice.org.uk.

### Study selection

Studies were included if: (1) the methodology of the study combined a literature search with expert panel opinion, (2) if the results of the study contained quality indicators on medication safety (for example: procedures on handling of (high alert) medications or rates of medication error), and (3) if any of the quality indicators were applicable to the medication preparation and administration phase in a hospital setting. Studies had to combine a literature search with expert panel opinion because this has been described as the most rigorous way to develop quality indicators [11, 16].

Two reviewers (MS and LV) independently selected studies from the search of the electronic databases based on title and abstract. The full texts of the studies selected in the first step were reviewed independently by the same reviewers. In case of disagreement, a third reviewer (HV) was involved to reach a consensus decision on inclusion. Also, the reference lists of the included studies were checked to identify any relevant studies that had not been found in the electronic search. To conclude, HV searched and reviewed the grey literature registries on January 19 2015, potential relevant hits were checked by MS.

### Data extraction and analysis

A structured data extraction form was used to describe the studies and quality indicators for medication preparation and administration process (S3 Appendix). The extracted information consisted of the applicable setting, a description of the quality indicator and/or the explanation/description of the numerator and denominator, and the type of quality indicator according to the structure, process and outcome framework of Donabedian. Two reviewers (MS and LV) independently completed the data extraction. Any disagreement between the reviewers was again resolved by consensus decision with a third reviewer (HV).

### Methodological assessment

For the methodological assessment of the quality of the included studies, we used the AIRE Instrument (Appraisal of Indicators through Research and Evaluation), which is a validated instrument developed to assess whether the aim and the organizational context of the quality indicators are clearly described and whether the quality indicators are evidence based [17]. The AIRE instrument contains 20 items, divided into four domains: purpose, stakeholder involvement, evidence and usage (S4 Appendix). Each item is scored on a four-point Likert scale ranging from 1 (strongly disagree; confident that the criterion has not been fulfilled or no information was available) to 4 (agree; confident that the criterion has been fulfilled). The AIRE instrument recommends a minimum of four independent reviewers for reliable scores. All authors (n = 7) independently appraised the included studies with the AIRE instrument. They represent a multidisciplinary group with backgrounds in internal medicine, surgery, pediatrics, pharmacy, nursing and quality of care. The AIRE Instrument was completed for a total set of quality indicators instead of for each quality indicator separately, because the studies only gave general information about the development of the total set of quality indicators and the evidence on which they were based. For each of the four domains, an independent standardized score can be calculated. However, we decided to use the individual item scores because we found them to be more informative. No guidance was available on the definition of a high or low score. For this study we arbitrarily defined individual item scores with a total between 7–13 as low, those between 14–20 as moderate and those between 21–28 as high. One author (MS) performed data entry and calculated the scores, while another (LV) cross checked the data entry and calculations.

## Results

### Search and study characteristics

**Fig 1:**
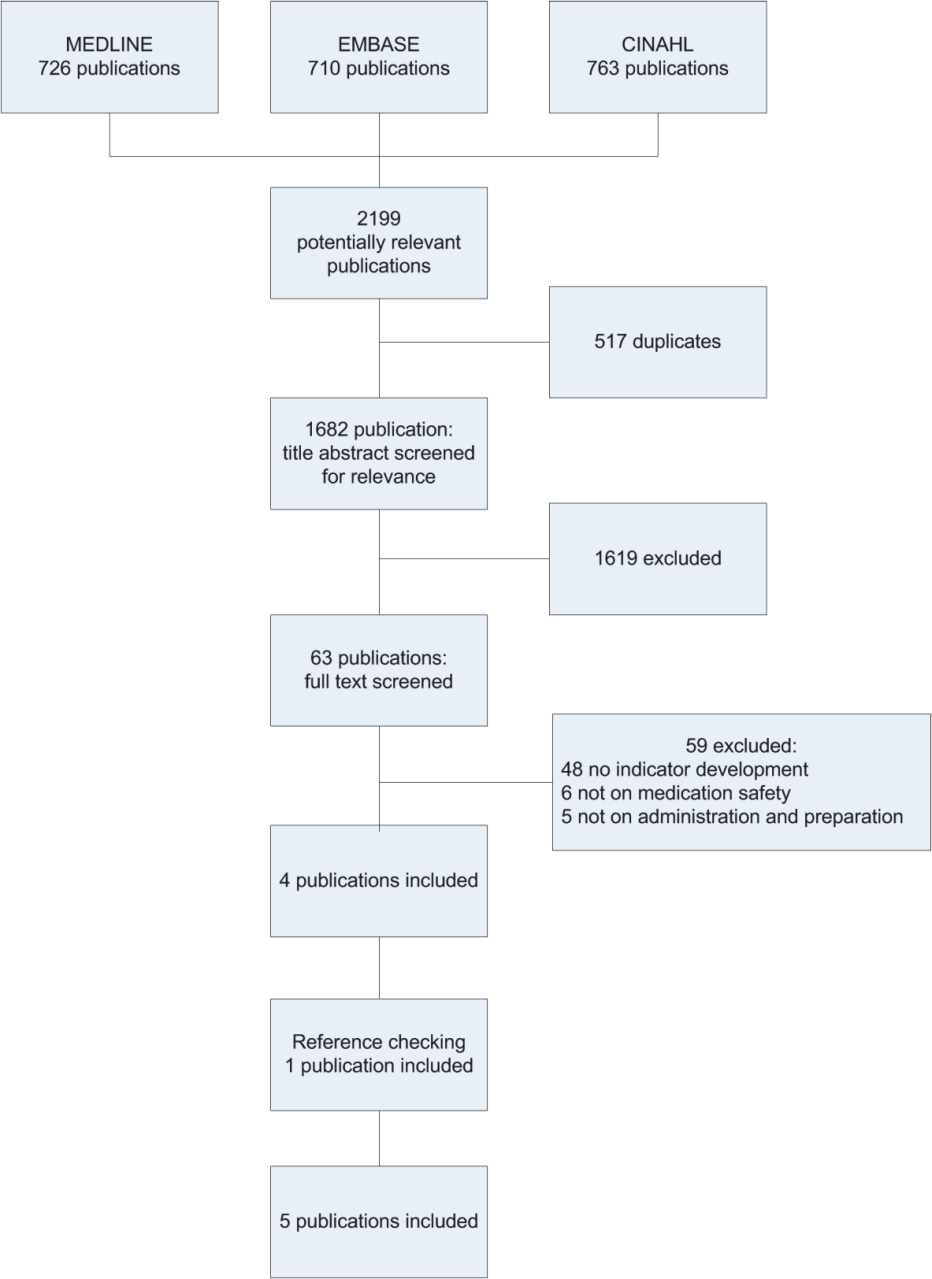
Flowchart of the literature search. The five included studies were published between 1995 and 2010 (Table 1). None of the studies had undertaken quality indicator development specifically for the nursing process of medication preparation and administration. Two studies (Cheng et al. and Nigam et al.) originated from Canada, and both aimed to develop quality indicators for medication safety [18, 19]. The Australian NSW TAG study was commissioned by the Department of Health, which used the data to publish a manual of medication safety indicators for hospitals [20]. This study was not listed as a scientific study; it was found through the reference search. The McLoughlin et al. study had a wider scope and was undertaken as part of the Organization for Economic Cooperation and Development (OECD) Quality Indicator Project, which aimed at developing an initial set of patient safety indicators. It involved several countries (Australia, Canada, the EU, Portugal, Spain and the United States) [21]. The QRC Advisor study was a study by the American Nurses Association that aimed to develop quality indicators for safety and quality of nursing care [22].

The electronic search identified 1682 potentially relevant studies. Based on their title and abstract, 63 studies were reviewed in detail. Four studies met the inclusion criteria. The main reason for excluding studies was the inability to meet the methodological inclusion criteria for a combined literature search and expert panel opinion. Checking the references of the included studies yielded one additional study, resulting in a total amount of five included studies (Fig 1 and S5 Appendix). The grey literature search did not result in any additional studies.

**Table 1:** Characteristics of studies and quality indicator sets.

*Author(s)*, *Year*, *Country*, *Reference*	*Population and setting*	*Number of indicators*: *Total and per Type*	*Indicator*
QRC Advisor, 1995, United States, [22]	Nursing indicators for the acute care setting	**Total: 1**	
		Structure:-	
		Process:-	
		Outcome: 1	Medication error rate
McLoughling et al, 2006, various countries, [21]	Patient safety indicators for OECD countries: Australia, Canada, the EU, Portugal, Spain and the United States	**Total: 1**	
		Structure: -	
		Process: -	
		Outcome: 1	Medication error
NSW TAG, 2007, Australia, [20]	Indicators for quality use in medicines in Australian hospitals	**Total: 5**	
		Structure: 1	Availability of concentrated potassium outside pharmacy
		Process: 4	Antibiotic therapy for surgical patients
			Postoperative pain management
			Adverse drug reaction
			Chemotherapy protocol
		Outcome:-	
Nigam et al, 2008, Canada, [19]	Medication safety indicators for both inpatient and outpatient settings in Canada.	**Total: 5**	
		Structure: -	
		Process: 5	Administering protocols for high alert prescription medications
			Verification of high alert prescriptions
			Machine readable coding systems for administration
			Differentiation of high alert prescription medication
			Monitoring and reducing Adverse Drug Events (ADEs) by assigning pharmacists on round
		Outcome:-	
Cheng et al, 2010,Canada, [18]	Medication safety indicators for public reporting in Canada.	**Total: 10**	
		Structure: 5	Top 10 medications
			Concentrated electrolytes
			Narcotic safety
			Incident reporting and analysis
			Prospective medication safety analysis
		Process: 2	Antibiotic prophylaxis
			Venous thromboembolism (VTE) prevention
		Outcome: 3	Medication incident types, harm or death incidents by type of error
			Medication incident rates, harm/death incidents per days of patient care
			Deaths associated with medication incidents

### Methodological assessment

According to the AIRE domains, the methodological quality of the included studies varied (Table 2). All the studies were clear on purpose, and most of them applied stakeholder involvement and used evidence reasonably. They described usage of the indicator in practice only occasionally [18–21]. The NSW TAG study published in 2007 had the highest scores, with high scores (21–28) in three of the four domains and moderate scores (14–20) in one domain [20]. Individual item scores were low (7–13) on item 8 (the indicator has been formally endorsed), item 11 (the supporting evidence has been critically appraised), item 14 (a strategy for risk adjustment has been considered and described) and item 17 (the indicator has sufficient discriminative power).

**Table 2:** AIRE item scores.

AIRE Scoring items	1. Purpose	2. Selection criteria	3. Organizational context	4. Quality domain	5. Healthcare process	6. Relevant professional groups included	7. Involvement	8. Formally endorsed.	9. Systematic search methods	10. Guideline recommendations or peer reviewed studies	11. Critical appraisal of supporting evidence	12. Description of numerator and denominator	13. Defined target patient population	14. Strategy for risk adjustment	15. Validity	16. Reliability	17. Discriminative power.	18. Practice pilot	19. Data collection efforts	20. Presenting and interpreting instructions
AIRE categories	1. Purpose, relevance and organizational context					2. Stakeholder involvement			3. Scientific evidence			4. Additional evidence, formulation and usage								
QRC Advisor, 1995	19	16	20	20	17	10	7	7	10	10	7	9	15	8	11	9	8	7	13	8
McLoughling 2006	**28**	**27**	**24**	**27**	18	11	11	14	11	11	10	19	17	9	**23**	14	9	17	**22**	12
NSW TAG 2007	**28**	**25**	**27**	**28**	**27**	**27**	**23**	**26**	16	**24**	17	**28**	**27**	17	**27**	**23**	**24**	**25**	**27**	**27**
Nigam et al, 2008	**24**	**21**	**22**	**28**	**27**	**28**	**26**	9	**21**	16	11	**26**	19	7	11	10	9	10	12	12
Cheng et al, 2010	**28**	**26**	**25**	**28**	**28**	20	16	12	**27**	20	17	19	**22**	10	**21**	15	9	15	**22**	16

High AIRE scores are in bold (21–28)

### Quality indicators

From the included studies, a total of 21 partly overlapping quality indicators were identified and categorized (Table 3): five structure indicators, eleven process indicators and five outcome indicators. Three structure indicators were related to the implementation of safety management systems: incident reporting and analysis, prospective medication safety analysis and the availability of a top 10 high alert medication list that are associated with harm or deaths (the original author classified the availability of a top 10 high alert medication list as an outcome indicator, but we classified it as a structure indicator because the list was derived from incident reports). Two studies reported structure indicators on the limitation of availability of high alert medications (concentrated electrolytes and narcotics). The process indicators can be divided into three categories: verification of prescriptions either by pharmacists or an electronic system, protocols for medications that are driven by specific administration regimes (high alert medications, chemotherapy, antibiotic and thromboprophylactic medications) and documentation of relevant medication related clinical information (adverse drug reactions and postoperative pain management). The only patient-relevant outcome indicators identified were in the category of adverse events, for which various definitions of MEs were used. Of the five definitions of MEs, four included only MEs that resulted in harm or death.

**Table 3:** Description of QIs by type of indicator.

*Category*	*Source*	*Indicator*	*Description and/or Numerator*, *Denominator of indicator*
**Structure indicators**			
Safety management	Cheng	Incident reporting and analysis	**Description**: Organization has a policy and process for reporting and analyzing medication incidents (yes/no).
	Cheng	Prospective medication safety analysis	**Description**: Organization conducts at least one medication safety related analysis per year (yes/no).
	Cheng	Top 10 medications	**Description**: List of top 10 medications associated with harm or death medication incidents.
Availability of high alert medication	NSW TAG	Concentrated potassium	**Description**: Percentage of medication storage areas outside pharmacy where potassium ampoules are available.
	Cheng	Concentrated electrolytes	**Description**: Concentrated electrolytes (concentrated potassium chloride, potassium phosphate and sodium chloride >0.9%) are removed from patient care areas (yes/no) (percentage of patient care areas where concentrated potassium vials are available).
	Cheng	Narcotic safety	**Description:** Three criteria: 1. Removal of hydromorphone ampoules or vials with concentration >2 mg/mL (except palliative care) (yes/no); 2. Removal of morphine ampoules or vials with concentrations >15 mg/ mL (yes/no); 3. Standardization and limitation of the number of parenteral narcotic (opioid) concentrations available (yes/no).
**Process indicators**			
Verification	Nigam	Monitoring and reducing adverse drug events by assigning pharmacists on round	Numerator**: Number of beds with daily pharmacist participation in interdisciplinary direct patient care.**
			Denominator**: All beds.**
	Nigam	Verification of high alert prescriptions	Numerator**: Number of prescriptions/medication orders for high-alert medications that are double-checked and documented by pharmacists before administration.**
			Denominator**: All prescriptions/medication orders for high alert medications.**
	Nigam	Machine readable coding systems for administration	Numerator**: Number of doses administered with machine readable code (bar codes).**
			Denominator: **All doses administered.**
Visual reminders	Nigam	Differentiation of high alert prescription medication	Numerator**: Number of high-alert prescriptions medications that are differentiated from other medications using flags, highlighting or some other system.**
			Denominator**: All high-alert prescription medications.**
Protocols	Nigam	Administering protocols for high alert prescription medications	Numerator**: Number of prescriptions/medication orders for high-alert medications using an administering protocol.**
			Denominator**: All prescriptions/medication orders for high-alert medications.**
	NSW TAG	Chemotherapy protocol	**Description**: Percentage of patients receiving cytotoxic chemotherapy whose treatment is guided by a hospital approved chemotherapy treatment protocol.
			**Numerator:** Number of patients prescribed chemotherapy whose treatment was guided by a hospital approved protocol.
			**Denominator:** Number of patients prescribed chemotherapy in sample.
	NSW TAG	Antibiotic therapy for surgical patients	**Description**: Percentage of patients undergoing specified surgical procedures that receive an appropriate prophylactic antibiotic regimen.
			**Numerator =** Number of patients undergoing specified surgical procedures that receive an appropriate prophylactic antibiotic regimen.
			**Denominator =** Number of patients who had a specified surgical procedure in sample.
	Cheng	Antibiotic prophylaxis	**Description**: Proportion of select surgical patients (coronary artery bypass graft, cardiac surgery, hip arthroplasty, knee arthroplasty, hysterectomy and vascular surgery) who receive prophylactic antibiotics.
	Cheng	Venous thromboembolism prevention	**Description**: Proportion of at-risk or eligible patients (undergoing major general or hip fracture surgery) who receive thromboprophylaxis.
Documentation of relevant medication related clinical information	NSW TAG	Postoperative pain management.	**Description**: Percentage of postoperative patients whose pain intensity is documented using an appropriate validated assessment tool.
			**Numerator**: Number of postoperative patients whose pain intensity is documented using an appropriate validated assessment tool.
			**Denominator**: Number of postoperative patients in sample.
	NSW TAG	Adverse drug reactions	**Description**: Percentage of patients whose known adverse drug reactions are documented on the current medication chart.
			**Numerator:** Number of patients whose known ADRs are documented on the current medication chart.
			**Denominato**r: Number of patients in sample.
**Outcome indicators**			
Adverse events	QRC Advisor	Medication error rate	Numerator and denominator**: not reported**
	McLoughling	Medication error	**Numerator**: Number of patient deaths, paralysis, coma, or other major permanent loss of function associated with a medication error.
			**Denominator**: The original developer of this indicator conceived of it as sentinel event indicator, i.e. it would reflect events that should never happen. Consequently, the original definition has no denominator. However, if the indicator were applied to the health system level, an appropriate denominator would have to be used to be able to compare rates across countries.
	Cheng	Medication incident types, harm or death incidents by type of error	**Description**: Frequency of medication incidents resulting in harm or death, categorized according to the type of incident (e.g., incorrect dose, incorrect medication, incorrect patient).
	Cheng	Medication incident rates, harm/death incidents per days of patient care	**Description**: Proportion of medication incidents that result in harm or death per days of patient care.
	Cheng	Deaths associated with medication incidents	**Description**: Proportion of total deaths in Ontario that are associated with medication incidents.

We mapped the identified quality indicator categories onto the 7 rights (Table 4). Almost all categories can be mapped onto “right drug” and “right dose”. The quality indicators on verification by a pharmacist and use of protocols for specific medication also address “right reason.” The electronic verification adds “right patient” and “right documentation.” The structure indicators in the category safety management cannot be mapped onto any of the 7 rights because these indicators are not specific for any of the rights. A prospective risk analysis or incident analysis, for example, can be on various topics in medication safety and can therefore be mapped onto any of the 7 rights. The definitions of the indicators in the outcome category adverse events are not specific enough to map onto any of the 7 rights.

**Table 4:** Mapping of indicator categories onto the ‘7 rights’

	Availability of high alert medication	Pharmacist Verification	Electronic verification	Medication protocols	Visual reminders	Documen-tation
**Right patient**			**✓**			
**Right drug**	**✓**	**✓**	**✓**	**✓**	**✓**	
**Right dose**	**✓**	**✓**	**✓**	**✓**	**✓**	
**Right time**						
**Right route**						
**Right reason**		**✓**		**✓**		
**Right documentation**			**✓**			**✓**

## Discussion

A total of 21 evidence-based quality indicators were identified for the nursing process of safe in-hospital medication preparation and administration according to the 7 rights. Since quality indicators are used for quality assessment and both internal and external transparency and benchmarking, it is important to use reliable and valid quality indicators with clear definitions. Our study indicates that only five studies used an evidence-based approach, with a combined literature search and expert panel opinion, for quality indicator development. We also found that the clinical implication and feasibility of the quality indicators were poorly studied or described. These are important aspects to consider, because if data collection for the indicator is difficult, this might result in poor or insufficient data and an unreliable indicator.

The extracted quality indicators can be categorized according to the structure, process and outcome framework. However, most quality indicators referred to the structure and process of care; the only outcome indicator found was adverse events expressed as patient harm or death.

The goals addressed by the 7 rights are only partly covered with the current quality indicators and mainly address “right drug” and “right dose.” Quality indicators on verification by a pharmacist and electronic verification rely on the availability of pharmacists and electronic systems, which are not common practice in every hospital. No quality indicators were found that address “right time” and “right route”. Electronic verification systems could also check on this, but the quality indicators we extracted did not include this function. The nurse-dependent “right time” is especially important for time-critical scheduled medications, such as those identified by the Institute for Safe Medication Practices (ISMP) [23]. These are medications for which maintenance doses that are administered with dosing schedule deviations of more than 30 minutes may cause harm or result in a substantially sub-optimal therapy or pharmacological effect. These include medications with a dosing schedule more frequent than every four hours, medications that must be administered apart from other medications and certain medications that require administration within a specified period of time before, after, or with meals (for example, rapid-, short-, or ultra-short-acting insulin and certain oral anti diabetic agents).

Four of the five outcome quality indicators we found use different definitions of “MEs resulting in harm and death.” Rates of MEs vary, depending on the detection method used as well as the definition of numerator and denominator. Due to this lack of uniformity, incidence rates are difficult to compare. A generally accepted definition of MEs that defines both scope and content is required [24]. Also, the MEs resulting in harm and death are only a small portion of all MEs and near misses; consequently this might be an opportunity to develop more specific definitions of MEs related to the 7 rights.

Overall, we conclude that evidence-based quality indicators for medication safety with clear definitions are scarce, but the identified quality indicators provide an excellent starting point for further development.

### Limitations

As demonstrated in this systematic review, little research has been done on quality indicators for the safety of medication preparation and administration. However, our restriction to include only indicators that are developed through an evidence based approach may have led to the exclusion of useful indicators that are developed with other approaches. For instance, in the NICE guideline on safe nurse staffing a clearly defined indicator on medication administration errors is reported, along with eight other indicators for safe staffing (e.g. falls, pressure ulcers, missed breaks, compliance with mandatory training) [25]. This indicator on medication administration errors is well described (numerator, denominator, data source) and has excellent face validity for medication preparation and administration. The interpretation of this indicator, on the other hand, requires caution because the observed rates are heavily influenced by the general reporting level of errors.

Also, we may have underestimated the quality of some studies. The methodological appraisal of the studies was based on information derived from the included studies, but the development process, in particular, was not always described in detail.

### Implications for research

The identified quality indicators focus mainly on specific medication procedures or protocols. Future development of quality indicators could therefore focus more on nursing-specific quality indicators for the critical phases of medication preparation and administration. The 7 rights can be used to identify topics on which quality indicators are needed. Our study shows that there is room for further development, especially on the right patient, right route, right time, right reason and right documentation. We recommend that future studies use the AIRE instrument when developing quality indicators, especially to overcome the currently underexposed areas such as formal endorsement, critical appraisal of the evidence, risk adjustment and sufficient discriminative power.

### Implications for practice

We found that most of the included studies had methodological flaws. However, the aphorism of evidence-based practice is to “use the best available evidence” [26] and from this perspective it is advisable to consider using the identified quality indicators. When selecting the indicators to be used locally, it is important for endorsers and users to critically review the quality indicators on acceptability, feasibility of acquiring the right data, reliability, sensitivity to change, and validity [11]. Also, adaptation of quality indicators to the local setting may be needed, because the medication process and system may vary between countries. Even when a quality indicator is evidence-based and clearly defined, hospital boards as well as professionals need to believe in the quality benefits to be gained in exchange for their efforts to collect all the necessary data, and it should be feasible for them to integrate it as much as possible in existing processes, electronic systems and data collections. Adaptation and review of quality indicators can be done, for instance, through surveys, interviews or focus groups. Periodic evaluation reports with insightful interpretation of the results can monitor performance, promote evidence-based quality improvement initiatives, and ultimately serve to evaluate effectiveness [27–29].

## Supporting Information

S1 Appendix PRISMA 2009 checklist. (PDF)

S2 Appendix Search strategies. (PDF)

S3 Appendix Data extraction form. (PDF)

S4 Appendix AIRE instrument. (PDF)

S5 Appendix PRISMA 2009 Flow diagram. (PDF)
