# Supplementary material for: Quality Indicators for Safe Medication Preparation and Administration: A Systematic Review
Source: PLoS One. 2015 Apr 17;10(4):e0122695. doi: 10.1371/journal.pone.0122695 (PMC4401721; doi:10.1371/journal.pone.0122695)
Supplement: S2 Appendix — (PDF) [file pone.0122695.s002.pdf]

Supporting information S2: MEDLINE, EMBASE and CINAHL search strategies

MEDLINE search

((((("Medical Errors"[Mesh:noexp] OR "Medication Errors"[MeSH] OR "Malpractice"[Mesh:noexp] OR "Medication Systems, Hospital"[Mesh] OR "Medication Systems"[MeSH Terms] OR medication safety[tiab] OR medication error*[tiab] OR error rate*[tiab] OR clinical error*[tiab] OR ((error*[tiab] OR safety[tiab]) AND (medication administered[tiab] OR medications administered[tiab] OR administered medication*[tiab] OR administer medication*[tiab] OR medication administration[tiab] OR medications administration[tiab] OR medication use[tiab] OR medication prescribe*[tiab] OR prescribed medication*[tiab])))) AND ("Safety"[MeSH] OR "Patient Safety"[Mesh] OR "Safety Management"[Mesh] OR "Morbidity"[Mesh] OR patient safety[tiab] OR medication-use safety[tiab] OR medication safety[tiab] OR drug safety[tiab] OR safe medication use[tiab] OR safety management[tiab] OR safety standard*[tiab] OR (safety[tiab] AND medication*[tiab]) OR (safety[tiab] AND indicator*[tiab]) OR (safety[tiab] AND hospital*[tiab]))) AND ("Quality Indicators, Health Care"[Mesh] OR "Health Status Indicators"[MeSH Terms] OR "Drug Monitoring"[Mesh] OR "Drug Administration Schedule"[Mesh:noexp] OR indicator*[tiab] OR quality care[tiab] OR system factors[tiab] OR system failure*[tiab] OR ((monitor*) AND (quality OR safety)))) AND ("Nurses"[MeSH] OR "Nurse's Role"[MAJR] OR "Nursing Care"[Mesh] OR "Nursing Process"[MeSH] OR "Education, Nursing"[Mesh] OR "Economics, Nursing"[MeSH] OR "Nursing Staff"[MAJR] OR "Hospitals"[Mesh] OR "Inpatients"[Mesh] OR "Patient Care Team"[MeSH] OR "Patient-Centered Care"[MeSH] OR "Total Quality Management/organization and administration"[MAJR] OR "Quality Assurance, Health Care/methods"[MAJR] OR nurse*[tiab] OR nursing[tiab] OR case manager*[tiab] OR hospitalization[mh] OR hospital[tiab] OR hospitals[tiab] OR hospitalized[tiab] OR hospitalised[tiab] OR hospitalization[tiab] OR hospitalisation[tiab] OR inpatient[tiab] OR inpatients[tiab]))) NOT (nursing home* OR "Nursing Homes"[Mesh] OR homecare[tiab] OR home care[tiab])

EMBASE Search

#	Query
1	medical error/ or medication error/ or malpractice/ or hospital organization/ or (medication safety or medication error* or error rate* or clinical error*).ti,ab,kw. or ((error* or safety) and (medication administered or medications administered or administered medication* or administer medication* or medication administration or medications administration or medication prescribe* or prescribed medication*)).ti,ab.
2	safety/ or *patient safety/ or morbidity/ or (patient safety or medication-use safety or medication safety or drug safety or safe medication or safety management or safety standard*).ti,ab. or ((safety and medication*) or (safety and indicator*) or (safety and hospital*)).ti,ab.

3	exp health status indicator/ or process monitoring/ or indicator*.ti,ab,kw. or (quality care or system factors or system failure*).ti,ab. or (monitor* and (quality or safety)).ti,ab.
4	nurse/ or *nurse attitude/ or nursing care/ or nursing process/ or nursing education/ or continuing education/ or health economics/ or *nursing staff/ or hospital patient/ or patient care/ or *total quality management/ or hospitalization/ or (nurse* or nursing or case manager* or hospital or hospitals or hospitalized or hospitalised or hospitalization or hospitalisation or inpatient or inpatients).ti,ab.
5	1 and 2 and 3 and 4
6	nursing home/ or (nursing home* or homecare or home care).ti,ab
7	5 not 6

CINAHL search

#	Query
S38	S36 NOT S35
S37	S36 NOT S35
S36	S6 AND S14 AND S20 AND S34
S35	MH "Nursing Homes+" OR (TI nursing home* OR AB nursing home* OR TI homecare OR AB homecare OR TI home care OR AB home care)
S34	S21 OR S22 OR S23 OR S24 OR S25 OR S26 OR S27 OR S28 OR S29 OR S30 OR S31 OR S32 OR S33
S33	TI nurse* OR AB nurse* OR TI nursing OR AB nursing OR TI case manager* OR AB case manager* OR TI hospital OR AB hospital OR TI hospitals OR AB hospitals OR TI hospitalized OR AB hospitalized OR TI hospitalised OR AB hospitalised OR TI hospitalization OR AB hospitalization OR TI hospitalisation OR AB hospitalisation OR TI inpatient OR AB inpatient OR TI inpatients OR AB inpatients
S32	(MH "Quality Assurance")
S31	(MH "Quality Improvement")
S30	(MH "Patient Centered Care")
S29	(MH "Multidisciplinary Care Team")
S28	(MH "Inpatients")
S27	(MM "Nursing Staff, Hospital")

S26	(MH "Nursing Costs")
S25	(MH "Education, Nursing")
S24	(MH "Nursing Process") OR (MH "Process Assessment (Health Care)")
S23	(MH "Nursing Care")
S22	(MH "Nurse Attitudes")
S21	(MH "Nurses")
S20	S15 OR S16 OR S17 OR S18 OR S19
S19	(TI monitor* OR AB monitor*) AND (TI quality OR AB quality OR TI safety OR AB safety)
S18	TI indicator* OR AB indicator* OR TI quality care OR AB quality care OR TI system factors OR AB system factors OR TI system failure* OR AB system failure*
S17	(MH "Drug Administration")
S16	(MH "Drug Monitoring")
S15	(MH "Health Status Indicators")
S14	S7 OR S8 OR S9 OR S10 OR S11 OR S12 OR S13
S13	(TI safety OR AB safety) AND (TI hospital* OR AB hospital*)
S12	(TI safety OR AB safety) AND (TI indicator* OR AB indicator*)
S11	(TI safety OR AB safety) AND (TI medication* OR AB medication*)
S10	(TI patient safety OR AB patient safety OR TI medication-use safety OR AB medication-use safety) OR (TI medication safety OR AB medication safety) OR (TI drug safety OR AB drug safety) OR (TI safe medication OR AB safe medication) OR (TI safety management OR AB safety management) OR (TI safety standard* OR AB safety standard*)
S9	(MH "Morbidity")
S8	(MH "Patient Safety")
S7	(MH "Safety")
S6	S1 OR S2 OR S3 OR S4 OR S5
S5	(TI error* OR AB error* OR TI safety OR AB safety) AND (TI medication administered OR AB medication administered OR TI medications administered OR AB medications administered OR TI administered medication* OR AB administered medication* OR TI administer medication* OR AB administer medication* OR TI medication administration OR AB medication administration OR TI medications

	administration OR AB medications administration OR TI medication prescribe* OR AB medication prescribe* OR TI prescribed medication* OR AB prescribed medication*)
S4	(TI medication safety OR AB medication safety) OR (TI medication error* OR AB medication error*) OR (TI error rate* OR AB error rate*) OR (TI clinical error* OR AB clinical error*)
S3	(MH "Health Facility Administration")
S2	(MH "Malpractice")
S1	(MH "Medication Errors") OR (MH "Health Care Errors") OR (MH "Treatment Errors")
