# Supplementary material for: Quality Indicators for Safe Medication Preparation and Administration: A Systematic Review
Source: PLoS One. 2015 Apr 17;10(4):e0122695. doi: 10.1371/journal.pone.0122695 (PMC4401721; doi:10.1371/journal.pone.0122695)
Supplement: S3 Appendix — (PDF) [file pone.0122695.s003.pdf]

Supporting information S3: Data extraction form

Reference (author, year of publication)	Objective	Study design	Participants	Type of indicator(s) (structure, process, outcome)	Description of Indicator(s)	Main findings
