# Supplementary material for: Quality Indicators for Safe Medication Preparation and Administration: A Systematic Review
Source: PLoS One. 2015 Apr 17;10(4):e0122695. doi: 10.1371/journal.pone.0122695 (PMC4401721; doi:10.1371/journal.pone.0122695)
Supplement: S4 Appendix — (PDF) [file pone.0122695.s004.pdf]

Supporting information S4: AIRE instrument

I. Purpose, relevance and organizational context

1. The purpose of the indicator is described clearly.
2. The criteria for selecting the topic of the indicator are described in detail.
3. The organizational context of the indicator is described in detail.
4. The quality domain the indicator addresses is described in detail.
5. The health care process covered by the indicator is described and defined in detail.

II. Stakeholder involvement

6. The group developing the indicator includes individuals from all relevant professional groups.
7. Considering the purpose of the indicator, all relevant stakeholders have been involved at some stage of the development process.
8. The indicator has been formally endorsed.

III. Scientific evidence

9. Systematic methods were used to search for scientific evidence.
10. The indicator is based on recommendations from an evidence based guideline or studies published in peer-reviewed scientific journals.
11. The supporting evidence has been critically appraised.

IV. Additional evidence, formulation, usage

12. The numerator and denominator are described in detail.
13. The target patient population of the indicator is defined clearly.
14. A strategy for risk adjustment has been considered and described.

15. The indicator measures what it is intended to measure (validity).

16. The indicator measures accurately and consistently (reliability).

17. The indicator has sufficient discriminative power.

18. The indicator has been piloted in practice.

19. The efforts needed for data collection have been considered.

20. Specific instructions for presenting and interpreting results.
