# Supplementary material for: Quality Indicators for Safe Medication Preparation and Administration: A Systematic Review
Source: PLoS One. 2015 Apr 17;10(4):e0122695. doi: 10.1371/journal.pone.0122695 (PMC4401721; doi:10.1371/journal.pone.0122695)
Supplement: S5 Appendix — (PDF) [file pone.0122695.s005.pdf]

Supporting information S5: PRISMA 2009 Flow Diagram

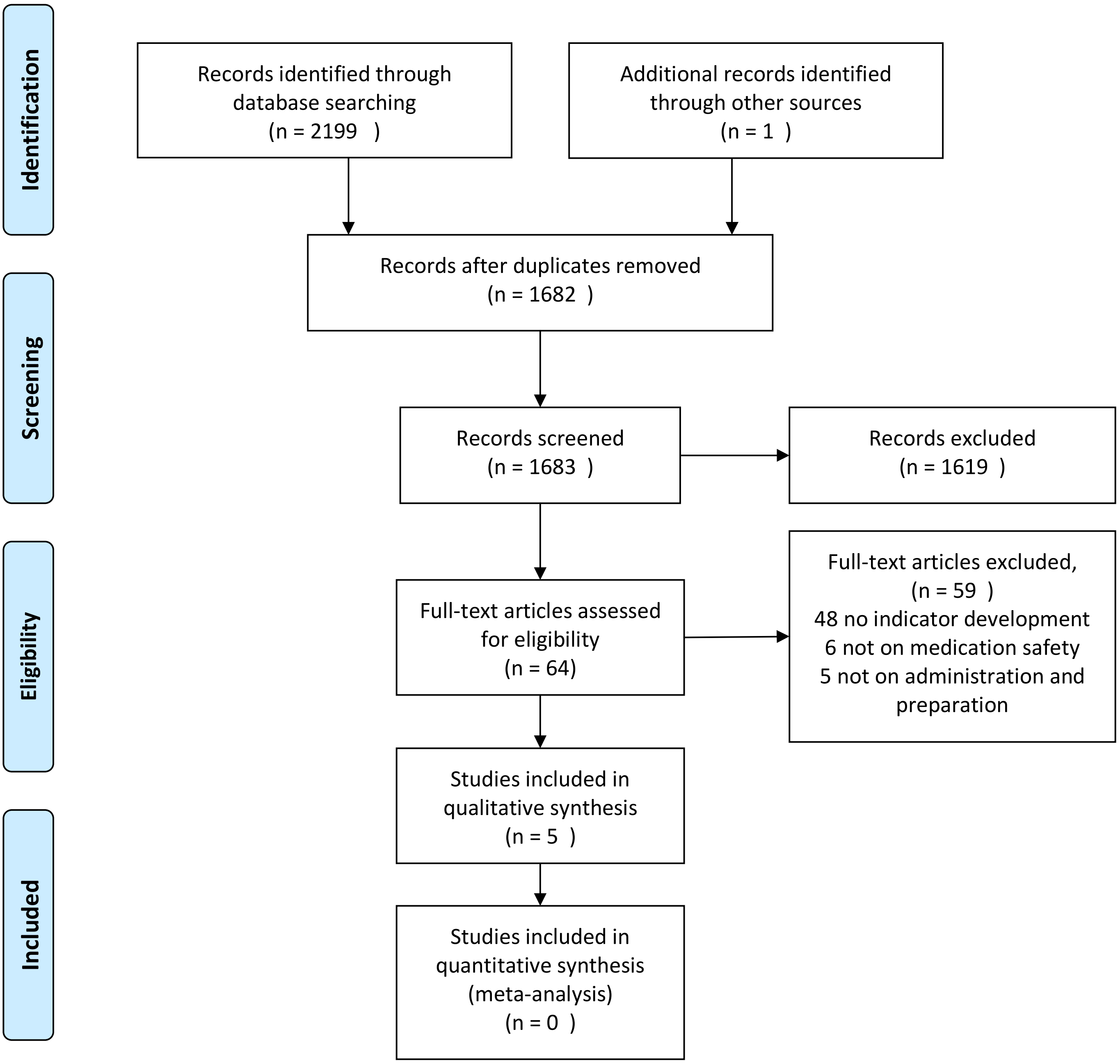

From: Moher D, Liberati A, Tetzlaff J, Altman DG, The PRISMA Group (2009). *P*referred *R*eporting *I*tems for *S*ystematic Reviews and *M*eta-*A*nalyses: The PRISMA Statement. PLoS Med 6(6): e1000097. doi:10.1371/journal.pmed1000097

For more information, visit www.prisma-statement.org.
